# The impact of genetic structure on sequencing analysis

**DOI:** 10.1186/s12919-016-0025-x

**Published:** 2016-10-18

**Authors:** Sneha Jadhav, Olga A. Vsevolozhskaya, Xiaoran Tong, Qing Lu

**Affiliations:** 1 Department of Statistics, Michigan State University, East Lansing, MI 48824 USA; 2 Department of Epidemiology and Biostatistics, Michigan State University, East Lansing, MI 48824 USA

## Abstract

**Background:**

Genome-wide association studies have made substantial progress in identifying common variants associated with human diseases. Despite such success, a large portion of heritability remains unexplained. Evolutionary theory and empirical studies suggest that rare mutations could play an important role in human diseases, which motivates comprehensive investigation of rare variants in sequencing studies. To explore the association of rare variants with human diseases, many statistical approaches have been developed with different ways of modeling genetic structure (ie, linkage disequilibrium). Nevertheless, the appropriate strategy to model genetic structure of sequencing data and its effect on association analysis have not been well studied.

**Methods:**

We investigate 3 statistical approaches that use 3 different strategies to model the genetic structure of sequencing data. We proceed by comparing a burden test that assumes independence among sequencing variants, a burden test that considers pairwise linkage disequilibrium (LD), and a functional analysis of variance (FANOVA) test that models genetic data through fitting continuous curves on individuals’ genotypes.

**Results:**

Through simulations, we find that FANOVA attains better or comparable performance to the 2 burden tests. Overall, the burden test that considers pairwise LD has comparable performance to the burden test that assumes independence between sequencing variants. However, for 1 gene, where the disease-associated variant is located in an LD block, we find that considering pairwise LD could improve the test’s performance.

**Conclusions:**

The structure of sequencing variants is complex in nature and its patterns vary across the whole genome. In certain cases (eg, a disease-susceptibility variant is in an LD block), ignoring the genetic structure in the association analysis could result in suboptimal performance. Through this study, we show that a functional-based method is promising for modeling the underlying genetic structure of sequencing data, which could lead to better performance.

## Background

Advancements in sequencing technology have enabled researchers to sequence exome regions or even the whole genome at affordable cost [1]. The emerging sequencing data facilitates the study of massive amounts of single nucleotide variants (SNVs), including both rare and common variants, for their potential role in complex human diseases. Although these studies hold great promise for identification of new disease-susceptibility variants, the extremely large number of SNVs brings significant challenge for association analysis. Conventional single-locus analysis suffers from low power because of the low frequency of SNVs and the issue of multiple testing. Grouping SNVs in a genetic region (eg, gene) could aggregate the association signal and alleviate the multiple testing issue, and therefore has been widely used in association analysis of sequencing data [2].

Various statistical methods have been proposed to group SNVs with or without considering the underlying genetic structure (ie, linkage disequilibrium [LD]). However, the impact of different strategies of modeling genetic structure on association results has rarely been investigated. If empirical evidence suggests that use of genetic structure in association analysis does not increase power, it gives us a basis for excluding this factor from statistical modeling. On the other hand, if it is important to consider LD among SNVs, then we need to investigate appropriate strategies for characterizing the underlying genetic structure. As an initial step to investigate this issue, we chose 3 tests with different ways of modeling LD between SNVs: (a) a weighted burden test assuming independence among SNVs (BT) [3]; (b) a weighted burden test considering pairwise LD (BTCOV) [4]; and (c) a functional analysis of variance (FANOVA) [5] test that considers LD among nearby loci and models the genotype profile of an individual as a continuous function.

## Methods

### Burden test

We consider a burden test developed by Madsen and Browning [3] that assumes independence among SNVs. The test summarizes the genetic score of all SNVs in a genetic region as ${\gamma}_i={\displaystyle \sum_{j=1}^L}\frac{g_{ij}}{w_j}$, where *L* is the number of SNVs, *g*_*ij*_ is the number of low-frequency alleles of the *j* ^*th*^ SNV for the *i* ^*th*^ individual. The weight is defined to emphasize the effect of rare variants with ${w}_j=\sqrt{n_j{q}_j\left(1-{q}_j\right)}$, where *n*_*j*_ is the number of controls and *q*_*j*_ is the minor allele frequency (MAF) of the *j* ^*th*^ SNV in controls. Analysis of variance (ANOVA) is then used to assess the association between summary genetic scores and the binary phenotype. Because the test simply adds the genotype of each SNV weighted by a function of its MAF, it does not consider LD between SNVs.

### Burden test that considers pairwise linkage disequilibrium

In addition to the above burden test, we also consider another type of burden test proposed by Schaid et al [4], which considers pairwise LD. We consider the following summary of genetic scores, ${S}_i={\displaystyle \sum_{j=1}^L}\frac{g_{ij}}{w_j}$, where *w*_*j*_ and *g*_*ij*_ are defined in the same manner as BT. However, unlike the conventional burden test, the test statistic of BTCOV is given by $T=\frac{{\left({\left(Y-\overline{Y}\right)}^{\hbox{'}}S\right)}^2}{{\left(Y-\overline{Y}\right)}^{\hbox{'}}{V}_s\left(Y-\overline{Y}\right)}$, where *Y* denotes the vector of disease status and $\overline{Y}$ is the mean disease status. *V*_*s*_ is the covariance matrix, where ${v}_{ii\hbox{'}}={\displaystyle \sum_{j=1}^L}{\displaystyle \sum_{j^{\prime }=1}^{L^{\prime }}}\frac{1}{w_j}\frac{1}{w_{j^{\prime }}}cov\left({g}_{ij},{g}_{i\hbox{'}j\hbox{'}}\right)$.

#### Functional analysis of variance

FANOVA considers LD by fitting a continuous function (curve) on the genotype data of an individual [5]. While various smoothing methods can be used to fit curves on individuals’ genotype data, we used cubic B-splines to fit the smooth functions [6]. By using cubic B-splines [5], we first fit *g*_*ik*_(*t*)*,* the smoothed function of genetic variants at the genomic position *t* for an individual *i* in the *k*th group. The FANOVA can then be used to compare the difference of curves in cases and controls. The FANOVA model can be written as:

$$ {g}_{ik}(t)={\mu}_k(t)+{\epsilon}_{ik}(t), $$

$$ {\epsilon}_{ik}(t)\to G.P\left(0,\gamma \right),\kern1em i=1,2,\kern0.5em \dots \kern0.5em ,{n}_k,\kern0.5em k=1,2 $$

where *i, k, n*_*k*_ and *t* denote the individual, the group (ie, case or control), the total number of individuals in the *k* ^*th*^ group, and the genomic position of a genetic variant, respectively. *G. P*(0, *γ*) stands for the gaussian process, where *γ* is the covariance function, *ϵ*_*ik*_ is the error term and *μ*_*k*_ is the mean function for the *k* ^*th*^ group. We test the following hypotheses:

$$ {H}_0:{\mu}_1(t)={\mu}_2(t)\ \forall\ t\ {H}_1:{\mu}_1(t)\ne {\mu}_2(t) $$

for some *t*.

Similar to ANOVA, the test statistic for the hypothesis can be constructed as,

$$ F=\frac{{\displaystyle \int }{\displaystyle {\sum}_{k=1}^2}{n}_k{\left(\hat{\mu_k}(t)-\hat{\mu}(t)\right)}^2dt/\left(2-1\right)}{{\displaystyle \int }{\displaystyle {\sum}_{k=1}^2}{\displaystyle {\sum}_{i=1}^{{\mathrm{n}}_{\mathrm{k}}}}{\left({g}_{ik}(t)-\hat{\mu_k}(t)\right)}^2dt/\left(n-2\right)}, $$

where $$ \hat{\mu_k}(t)={\displaystyle \sum_{i=1}^{n_k}}{g}_{ik}(t){n_k}^{-1} $$ and $$ \hat{\mu}(t)={\displaystyle \sum_{k=1}^2}{\mathrm{n}}_{\mathrm{k}}\hat{\mu_k}(t){\mathrm{n}}^{-1} $$.

### Simulation

Simulations were conducted to compare the performance of the 3 methods using a simulation model specified here as well as on the Genetic Analysis Workshop 19 (GAW19) simulated phenotype data. First, we selected a subset of 142 unrelated individuals from the GAW19 family-based sequencing data. For each replicate, we randomly chose a 30-kb segment from the 1.4 Mb region (chromosome 3: 33100124 to 34539295). From each segment, we randomly selected a specified proportion of SNVs (between 1 and 50 % as given in Tables 1 and 2) as disease-associated SNVs. A logistic regression model was then applied to the selected SNVs to simulate a binary phenotype. In the simulation, we considered 2 types of effects, bidirectional effects and unidirectional effects, by randomly generating the regression coefficients from N (0, 1) and N (2, 1), respectively. One thousand replicates were simulated for each scenario for power and type I error estimation. For FANOVA, we used the penalized cubic B-splines to determine the smoothness of the functions. The smoothing parameter was determined by using the generalized cross validation.

**Table 1:** Power comparison of 3 tests in the case of unidirectional effects

Test	Proportion of causal variants							
	0.01	0.05	0.1	0.15	0.2	0.25	0.3	0.5
BT	0.343	0.617	0.714	0.766	0.759	0.776	0.793	0.781
BTCOV	0.339	0.615	0.712	0.767	0.755	0.780	0.792	0.794
FANOVA	0.398	0.700	0.764	0.808	0.807	0.814	0.814	0.744

**Table 2:** Power comparison of 3 tests in the case of bidirectional effects

Test	Proportion of causal variants							
	.01	.05	0.1	0.15	0.2	0.25	0.3	0.5
BT	0.208	0.508	0.585	0.621	0.665	0.678	0.703	0.683
BTCOV	0.200	0.509	0.579	0.618	0.663	0.668	0.698	0.680
FANOVA	0.217	0.597	0.683	0.732	0.765	0.799	0.809	0.815

The above simulations only evaluated 1 genetic region. To investigate the performance of the 3 methods on regions with different genetic structures, we also applied them to the subset of 142 unrelated samples from the GAW19 family-based simulated data, of which 24 samples are cases. This data consists of 294 genes, including *THRA* and *RELB*, which were simulated to be associated with the hypertension phenotypes. For the association analysis, hypertension (HTN1) from the first simulation out of the 200 simulations was used.

## Results

Type I error rates of the 3 tests are well controlled at the 0.05 level (0.046 for BT, 0.044 for BTCOV, and 0.047 for FANOVA). As we observe from Table 1, power of the 3 tests increases as we increase the proportion of disease-associated variants. Overall, FANOVA has better or comparable performance to BT and BTCOV, while BTCOV obtains similar power to BT. The same conclusion also holds when the effects are bidirectional (see Table 2). We also observe that the power of the 3 tests is slightly lower in the case of bidirectional effects than in the case of unidirectional effects.

Table 3 summarizes the top 10 genes with the smallest *p* values from the association analysis. Consistent with the result from simulations, we find that in general FANOVA attains smaller *p* values, while the *p* values of BT and BTCOV were comparable.

**Table 3:** Summary of the 10 genes with the smallest *p* values from the association analysis

Gene	Test		
	BT	BTCOV	FANOVA
*SUMF1*	8.75E-05	8.43E-05	1.31E-05
*RELB*	6.57E-02	7.08E-02	4.35E-04
*HIF3A*	2.12E-02	2.19E-02	4.34E-03
*THRA*	1.85E-02	9.68E-03	3.62E-02
*TFDP1*	1.50E-02	1.13E-02	1.95E-02
*PROK2*	1.23E-02	1.32E-02	1.27E-01
*POLR2A*	2.10E-02	2.25E-02	1.31E-02
*CD1C*	2.76E-02	1.42E-02	5.27E-02
*CCL24*	2.24E-02	2.71E-02	1.92E-02
*MAP3K6*	8.72E-02	8.45E-02	3.27E-02

## Discussion

**Fig. 1:**
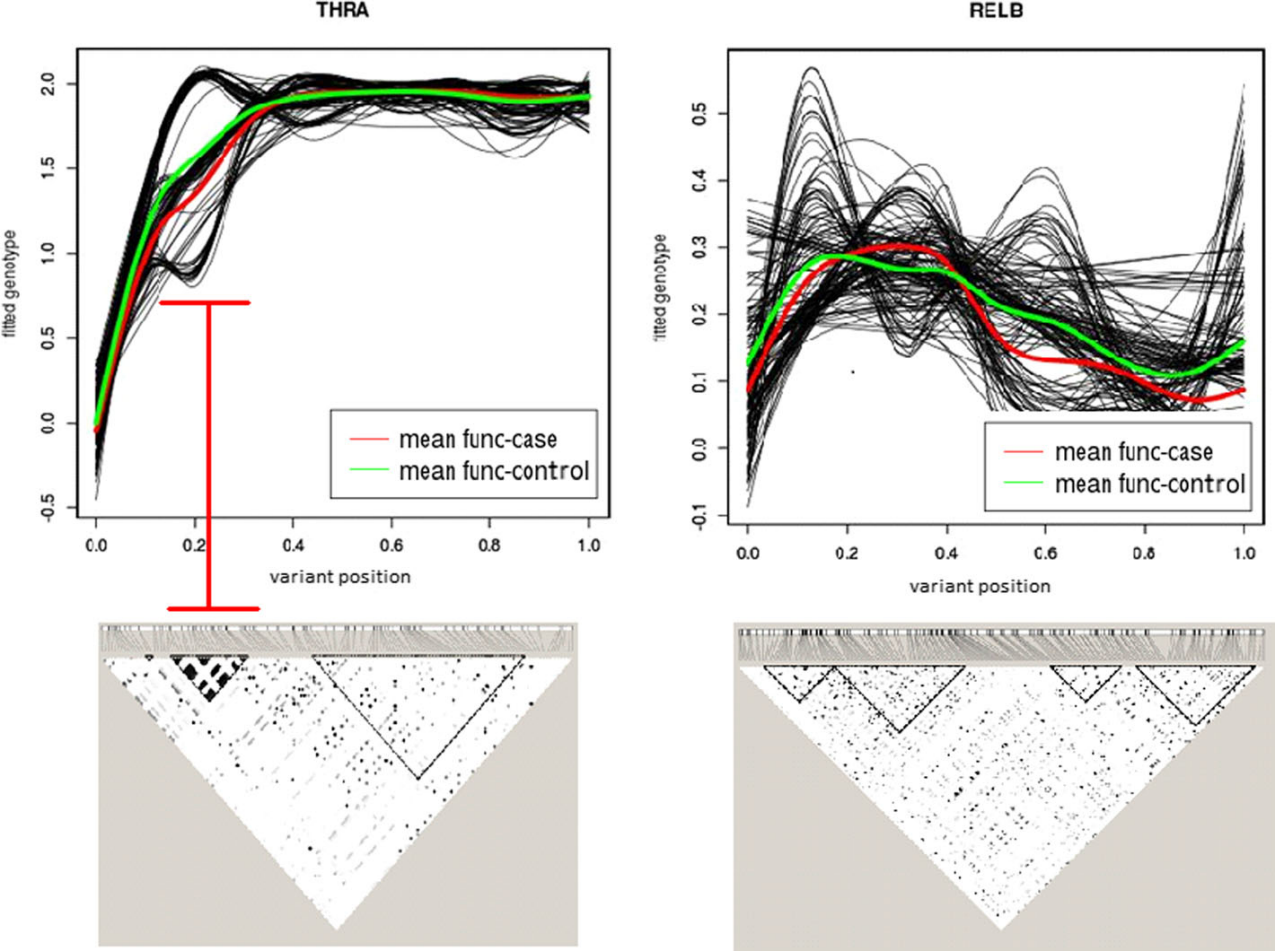
Fitted smooth function plots and LD plots for *THRA* and *RELB.* LD plots were obtained by using the software Haploview, in which triangles indicate LD blocks. The black curves are fitted smooth functions for all individuals. The red and green curves indicate the mean functions of cases and controls, respectively. The red structure on the left side of the figure indicates that a region harboring a possible association corresponds to a LD block

Through this study, we find that overall BT and BTCOV have comparable performance. However, for 1 gene, *THRA*, BTCOV attains a lower *p* value than the other 2 tests. In the follow-up analysis, we observe a small LD block in this gene (Fig. 1). The plot of the fitted genotype curves reveal the association happens to lie in that LD block. Therefore, BTCOV, which models the LD pattern, outperforms the other 2 tests. The plot also shows that FANOVA is able to capture the LD block. Nevertheless, the effects in the LD block are largely unidirectional, which is in favor of burden tests.

Variants may have more complex structure than pairwise LD. Hence, in most cases we find that FANOVA has comparable or better performance than the other 2 tests. For example, FANOVA attained a lower *p* value than the other 2 tests in the analysis of *RELB*. From Fig. 1, we observe that FANOVA not only captures the LD structure but also bidirectional effects, which are indicated by the crossing of the curves for cases and controls.

## Conclusions

Our observations indicate that the performance of tests depends on the underlying genetic structure; hence, ignoring LD in the association analysis may not be ideal. It is advisable to use function-based approaches to explore and model the genetic structure. As illustrated by Fig. 1, the plot of the fitted functional curves provides a great way to explore the genetic structure. The disease-associated regions can also be visualized in this plot. If the underlying genetic structure tends to be complex (eg, having multiple LD blocks with different effects), it is also advisable to use function-based approaches, such as FANOVA, to adequately model the sequencing data.
